# Sitting ducklings: Timing of hatch, nest departure, and predation risk for dabbling duck broods

**DOI:** 10.1002/ece3.5146

**Published:** 2019-04-16

**Authors:** Sarah H. Peterson, Joshua T. Ackerman, Mark P. Herzog, C. Alex Hartman, Rebecca Croston, Cliff L. Feldheim, Michael L. Casazza

**Affiliations:** ^1^ U.S. Geological Survey, Western Ecological Research Center Dixon Field Station Dixon California; ^2^ California Department of Water Resources Suisun Marsh Program West Sacramento California

**Keywords:** *Anas cyanoptera*, *Anas platyrhynchos*, imprinting, *Mareca strepera*, nest depredation, nest exodus, predation

## Abstract

For ground‐nesting waterfowl, the timing of egg hatch and duckling departure from the nest may be influenced by the risk of predation at the nest and en route to wetlands and constrained by the time required for ducklings to imprint on the hen and be physically able to leave the nest. We determined the timing of hatch, nest departure, and predation on dabbling duck broods using small video cameras placed at the nests of mallard (*Anas platyrhynchos*; *n* = 26), gadwall (*Mareca strepera*; *n* = 24), and cinnamon teal (*Anas cyanoptera*; *n* = 5). Mallard eggs began to hatch throughout the day and night, whereas gadwall eggs generally started to hatch during daylight hours (mean 7.5 hr after dawn). Among all species, duckling departure from the nest occurred during daylight (98%), and 53% of hens typically left the nest with their broods 1–4 hr after dawn. For mallard and gadwall, we identified three strategies for the timing of nest departure: (a) 9% of broods left the nest the same day that eggs began to hatch (6–12 hr later), (b) 81% of broods left the nest the day after eggs began to hatch, and (c) 10% of broods waited 2 days to depart the nest after eggs began to hatch, leaving the nest just after the second dawn (27–42 hr later). Overall, eggs were depredated at 10% of nests with cameras in the 2 days prior to hatch and ducklings were depredated at 15% of nests with cameras before leaving the nest. Our results suggest that broods prefer to depart the nest early in the morning, which may best balance developmental constraints with predation risk both at the nest and en route to wetlands.

## 1 INTRODUCTION

After eggs hatch, dabbling duck hens nesting in upland fields often move their broods to nearby wetlands (Amundson & Arnold, 2011; Chouinard & Arnold, 2007; Krapu, Pietz, Brandt, & Cox, 2006; Mauser, Jarvis, & Gilmer, 1994a), to provide ducklings with access to fresh water and food (Kear, 1965). Predators consume significant proportions of waterfowl eggs and young birds (Ackerman, Eadie, Loughman, Yarris, & McLandress, 2003; Chouinard & Arnold, 2007; Cowardin, Gilmer, & Shaiffer, 1985; Croston et al., 2018; Sargeant & Raveling, 1992), and predation is the primary determinant of egg and duckling survival (Chouinard & Arnold, 2007; Korschgen et al., 1996; Mauser, Jarvis, & Gilmer, 1994b; Sargeant & Raveling, 1992). Noise and movement may increase as eggs begin to hatch (Afton & Paulus, 1992; Boyd & Fabricius, 1965), which could alert predators and increase predation risk at the nest. If so, minimizing time at the nest after hatch may be advantageous, and consequently, timing egg hatch and duckling departure from the nest to minimize predation risk may be advantageous to waterfowl. Departure from the nest as soon as possible during daylight hours, when key predators are less active (Croston et al., 2018), might be one way to reduce the risk of predation; however, this timing may conflict with the time needed for ducklings to develop motor skills and imprint upon their mother (Fabricius, 1964). Visual and auditory imprinting on the mother by offspring is important for ducklings to maintain contact with a hen as they move through densely vegetated upland and wetland habitats (Fabricius, 1964).

Duckling broods also are vulnerable to predation during overland movements between nests and wetlands (Chouinard & Arnold, 2007; Mauser et al., 1994b), and the risk of predation during these movements may differ from predation risk on ducklings at the nest. Ducklings may be more visible during these overland movements than at the nest or in wetland habitats, increasing their vulnerability to predation by a suite of mammalian, avian, and reptilian predators during this transition. Consequently, decreasing predation risk to ducklings may entail reducing the time at the nest after hatch while at the same time leaving the nest at a time that would minimize predator contact during the period of transit between the nest and wetlands.

Although the risk of predation is likely a primary driver of the timing of duckling departure from the nest, the maturation of motor abilities and imprinting may also influence the timing of nest departure (Fabricius, 1964). Imprinting is an important developmental step after hatch for young birds (Afton & Paulus, 1992; Hess, 1959), particularly in nidifugous species that leave the nest shortly after hatch, and facilitates simultaneous movement of a hen and her offspring from the nest to subsequent habitats. A critical period of visual and auditory imprinting may occur 13–16 hr after hatch, when ducklings most effectively imprint on the hen (Bjärvall, 1967; Fabricius, 1964; Hess, 1959). Departure from a nest prior to a critical imprinting period is possible but may impair the ability of ducklings to follow and maintain contact with the hen (Fabricius, 1964); ducklings that are not with a hen during overland movements have decreased survival (Krapu, Dwyer, & Luna, 1991). After hatch, duckling feathers need to fully dry (within 4 hr of hatch; Kear, 1965) and duckling motor development reaches a point where they are physically ready to leave the nest (Fabricius, 1964). Thus, the timing of departure from a nest may be influenced by a combination of developmental factors which may interact with selective pressure from predation, both at the nest and during transit to brood ponds.

We examined the timing of egg hatch, the timing of nest departure, and predation on eggs and ducklings, during this critical period between hatch and departure, for three species of dabbling ducks. Specifically, we used videography to determine whether (a) the start of egg hatch and (b) duckling departure from the nest occurred randomly throughout the day or if these events were more likely to occur during specific times of day. We identified predators at nests and assessed whether nests containing recently hatched ducklings were more vulnerable to predation than active nests with eggs during the 2 days prior to hatch, to assess whether sound and movement associated with the presence of ducklings themselves increases the likelihood of nest depredation.

## 2 METHODS

### 2.1 Study area

We studied nesting dabbling ducks on the Grizzly Island Wildlife Area (California, USA; 38.141°N, 121.970°W), within Suisun Marsh, an important breeding site for dabbling ducks in California, USA (Ackerman, Blackmer, & Eadie, 2004; McLandress, Yarris, Perkins, Connelly, & Raveling, 1996). The majority of nesting sites are found within an 800‐ha block of upland nesting fields, and hens and ducklings must move over land from upland nesting fields to adjacent wetland habitats suitable for brood rearing (Ackerman, 2002; McLandress et al., 1996). Nests are typically located within 1,000 m of the nearest potential wetland (some nests are within 100 m), although the proximity of nests to wetland habitats changes over the course of the nesting season, as landowners and state wildlife managers manage water delivery and affect water levels in wetland habitats.

### 2.2 Nest monitoring

We used standard nest‐searching techniques, modified from McLandress et al. (1996), to find mallard (*Anas platyrhynchos*), gadwall (*Mareca strepera*), and cinnamon teal (*A. cyanoptera*) nests in upland fields from March to July, 2015 to 2017. We searched upland fields every 3 weeks with a rope pulled between 2 all‐terrain vehicles (ATVs), and we visited previously discovered nests weekly to monitor nest development and fate. We determined incubation stage by candling all eggs (Weller, 1956) and placed small video surveillance cameras (EZspy Cam, Los Angeles, CA, USA; IR Nightvision Camera #ENC‐102NRA, Day Night Lipstick Camera with invisible 950‐nm infrared LED and 3.6‐mm lens) at a subset of nests ≥6 days in incubation, following the protocol described in Croston et al. (2018). Each deployed camera remained at the nest until the nest was no longer active, and cameras were retrieved from successful nests after ducklings had departed. Our estimates of nest fate did not differ between nests monitored with standard techniques and camera‐monitored nests (Croston et al., 2018).

### 2.3 Timing of egg hatch and duckling departure

We determined the time at which we detected the first duckling fully emerged from an egg (hereafter start of hatch) and the time of duckling departure from the nest using the videos recorded at all hatched nests (Table 1). To be included in analyses, video footage needed to cover the period of duckling hatch until the final fate of the brood could be determined (predation or departure from the nest).

**Table 1:** Sample sizes of mallard (*Anas platyrhynchos*), gadwall (*Mareca strepera*), and cinnamon teal (*Anas cyanoptera*) nests that had small video cameras deployed to determine the timing of the start of hatch (the time at which the first duckling fully emerged from an egg), end of hatch (the time at which the last duckling was fully emerged from an egg), duckling departure from the nest, egg depredation in the ~2 days prior to expected hatch, and duckling depredation at the nest

Species	Start of hatch	End of hatch	Duckling departure	Predation on eggs	Predation on ducklings
Mallard	26	8	24	3	4
Gadwall	24	19	23	3	4
Cinnamon teal	5	2	5	0	0
Total nests	55	29	52	6 (of 60)^a^	8 (of 55)

Note

This study took place at the Grizzly Island Wildlife Area (California, USA; 38.141°N, 121.970°W), within Suisun Marsh, 2015–2017.

a Includes nests that did not hatch because of predation.

Once we determined the start of hatch time, the video footage was watched forward continuously until departure of all ducklings and the hen from the nest. The end of hatch was recorded if the observer saw the last duckling emerge from an egg or determined that there were no longer any more viable eggs left to hatch. The end of hatch was more difficult to ascertain than the start of hatch, due to visual obstruction by the hen and already‐hatched ducklings; thus, we only recorded an estimate for the end of hatch for a smaller subset of nests (Table 1). Using video cameras to estimate the end of hatch may have underestimated the duration of time that the brood was fully hatched at the nest; however, this would provide a minimum and, consequently, a conservative estimate for the time that the brood remained at the nest after hatch had ended and prior to departure. We recorded the departure from the nest as the date and time the hen left the nest with her ducklings.

### 2.4 Egg and duckling depredation

For visits to the nest by potential predators during the time from the start of hatch to duckling departure, we recorded predator type to the lowest taxonomic level possible and the date and time that a potential predator appeared and disappeared from the camera frame. The numbers of eggs and ducklings present and alive at the start and end of each predator appearance on camera were also recorded as well as the number of ducklings that were killed or taken away by the predator. Following Croston et al. (2018), we grouped observations of the same predator type into separate predation bouts only if the time elapsed between successive entrances into the camera frame exceeded 1 hr.

To test whether predation was more likely at nests just prior to or just after the start of hatch, we examined all nests with cameras (including those that did not hatch because of predation) that were still viable ~2 days prior to the expected hatch date. We watched the video footage during the ~2‐day period prior to hatch to identify predators, and we recorded the date, time, and number of eggs that were present at the start and end of each predation bout. The expected hatch date was estimated using both the clutch completion date and the mean estimated incubation stage during the last visit by investigators.

### 2.5 Statistical analysis

#### 2.5.1 Standardizing time of day

Once we had times associated with the start of hatch and duckling departure from the nest, we standardized all times of day to the onset of daylight, with nautical dawn as time 0. We used the GeoLight package in R (Lisovski & Hahn, 2012) to calculate the solar zenith angle (°) of each nest, using latitude and longitude, at the corresponding date and time over the course of the nesting season. The occurrence of every event was then classified as night, day, or twilight (period between nautical dawn and sunrise or between sunset and nautical dusk) based on the solar zenith angle (day <90°, twilight ≥90° and ≤102°, and night >102°). First light (nautical dawn; hereafter dawn) for the events in our study area over the course of the nesting season ranged from 04:33 to 05:20 hr local time and sunrise ranged from 05:44 to 06:21 hr local time. Sunset ranged from 19:51 to 20:34 hr and last light (nautical dusk; hereafter dusk) ranged from 20:52 to 21:45 hr local time. For visualization purposes in figures, all times were standardized so that 05:00 hr local time was shown as dawn (time 0) and 21:00 hr was shown as dusk; however, all statistical analyses were conducted using time relative to nautical dawn.

#### 2.5.2 Timing of egg hatch and duckling departure

To examine the timing of the start of hatch and duckling departure from the nest, we used circular statistics in the package CircStats (Lund & Agostinelli, 2018) in R v. 3.5.0 (R Core Team, 2017). We transformed the time since dawn to radians by taking the decimal hour of each event (0–23), dividing by 24, and then multiplying by 2*π* (Zar, 1999). We tested for differences in the distribution of both the timing of the start of hatch and duckling departure between mallard and gadwall. If there were no significant differences in the distributions between species, then we pooled the species and ran Watson's tests to test the null hypothesis of circular uniformity for (a) the timing of the start of hatch and (b) the timing of duckling departure from the nest, relative to dawn. We did not have a large enough sample size to include cinnamon teal in circular statistical analyses. When we present mean values, we use the back‐transformed mean of the circular radian hour.

Additionally, we tested whether ducklings hatched and broods departed nests preferentially during daylight. To do so, we used a chi‐squared goodness‐of‐fit to compare the observed counts of events occurring during daylight (dawn to dusk) versus events completely at night with the expected counts. We observed a median 16.8 hr of daylight on the day of hatch for the nests in this study; consequently, 70% of events should occur during daylight if events occurred randomly with respect to time of day.

We compared the length of time that ducklings remained in nests after the first duckling in each nest was observed using a general linear model with species as a factor (mallard, gadwall, and cinnamon teal). Additionally, we identified three departure strategies for mallard and gadwall, based on timing, and used a general linear model to assess potential species differences in time spent at the nest after the start of hatch, while accounting for the different departure strategies. The first strategy included ducklings that left the nest during the same day the first duckling hatched (hereafter “same day” departure), the second strategy included ducklings that stayed at the nest through the remainder of the day on which the first duckling hatched and departed during the next day after dawn (hereafter “next day” departure), and the third strategy included the ducklings that waited until after two consecutive dawns to depart the nest (hereafter “after second dawn” departure). We used post hoc pairwise *t* tests to test for differences between species.

#### 2.5.3 Egg and duckling depredation

We compared the frequency of nests with eggs that were depredated just prior to hatch to the frequency of nests with depredated ducklings, using a chi‐squared test.

Values reported in the *Results* section are either means ± standard deviation (*SD*) or means ± 95% confidence intervals (figures). Analyses were conducted in R v. 3.5.0 (R Core Team, 2017), and *α* was set at *p* = 0.05.

## 3 RESULTS

### 3.1 Timing of egg hatch and duckling departure

**Figure 1:**
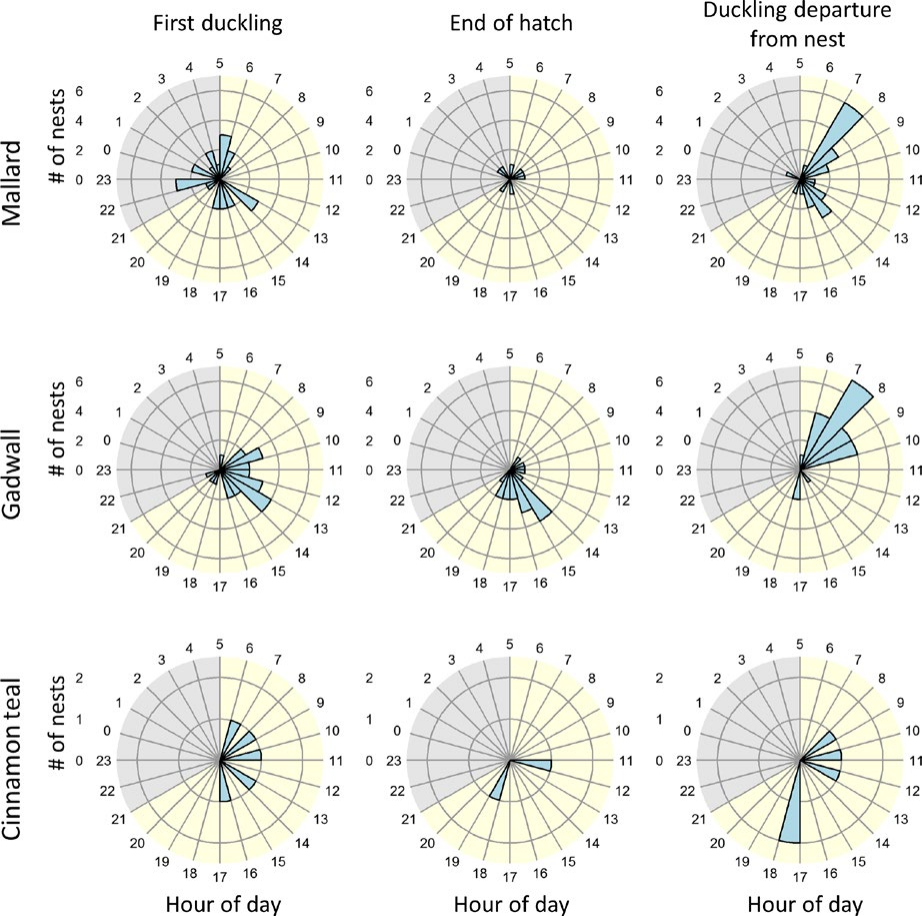
The timing of the first duckling to hatch (left column; *n* = 55 nests), the last duckling to hatch (middle column; *n* = 29 nests), and duckling departure from the nest (right column; *n* = 52 nest) was determined using video cameras deployed at mallard (*Anas platyrhynchos*; top row), gadwall (*Mareca strepera*; middle row), and cinnamon teal (*Anas cyanoptera*; bottom row) nests. All times were standardized so that 05:00 hr local time is dawn in this figure, although statistics were conducted using time relative to nautical dawn. Daylight is shaded in yellow, and night is shaded in gray. Note that the *y*‐axis scale is different for cinnamon teal. This study took place at the Grizzly Island Wildlife Area (California, USA; 38.141°N, 121.970°W), within Suisun Marsh, 2015–2017

Mallard (*n* = 26 nests) and gadwall (*n* = 24 nests; Table 1) ducklings did not start to hatch at the same time of day (Watson's *U* ^2^ = 0.45, *p* < 0.001; Figure 1). The start of hatch (the time at which the first duckling fully emerged from an egg) by mallard ducklings was randomly distributed between day and night periods, as there was a lack of support for rejection of the null hypotheses of circular uniformity (Watson's test = 0.08, *p* > 0.10). Furthermore, the observed count of nests that started to hatch during daylight and night hours was no different than the expected count (*χ* ^2^ < 0.08, *df* = 1, *p* = 0.77). In contrast, gadwall did not demonstrate circular uniformity in the hatch start time (Watson's test = 0.56, *p* < 0.01) and the start of hatch typically occurred during daylight (*χ* ^2^ = 6.02, *df* = 1, *p* = 0.01). On average, gadwall started to hatch at a mean of 7.5 hr after dawn and 80% of nests were observed starting to hatch between 3 and 10 hr after dawn (~08:00–15:00 hr local time). All cinnamon teal started to hatch during daylight between dawn and 12 hr after dawn (Figure 1).

Ducklings typically departed nests during daylight hours from 0.9 to 13.8 hr after dawn (*n* = 52; Table 1), with only one brood departing the nest during the night (Figure 1). We did not observe a difference in the distribution of duckling departure times for mallard (*n* = 24) or gadwall (*n* = 23; Watson's *U* ^2^ = 0.15, *p* > 0.10); thus, we examined circular uniformity for both species together. The circular distribution for the hour of mallard and gadwall duckling departure from the nest was not uniform (Watson's test = 1.18, *p* < 0.01), with ducklings preferentially leaving the nest during the daylight hours (*χ* ^2^ = 11.22, *df* = 1, *p* < 0.001). Mallard and gadwall ducklings most frequently departed the nest between 2 and 3 hr after dawn, with a mean departure from the nest 4.3 hr after dawn. Cinnamon teal ducklings (*n* = 5 broods), on average, departed the nest 8.2 hr after dawn. Three nests used in the analysis of the start of hatch were omitted from the analysis of nest departure because in one nest, the sole surviving duckling left the nest while the hen continued to incubate inviable eggs; in another nest, the hen and ducklings were killed by a predator; and in the third nest, all of the ducklings were killed by a predator.

**Figure 2:**
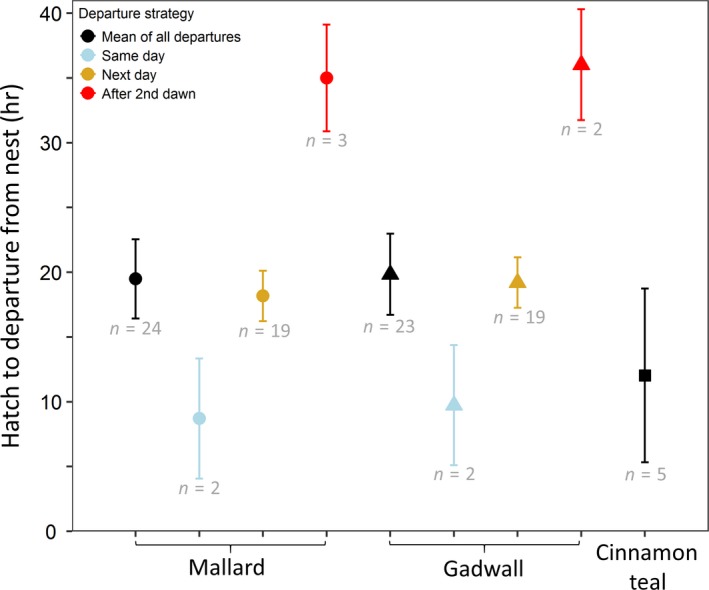
The duration of time at the nest between the first duckling to hatch and departure from the nest by mallard (*Anas platyrhynchos*; circle), gadwall (*Mareca strepera*; triangle), and cinnamon teal (*Anas cyanoptera*; square) ducklings, shown using model‐generated least squares means with 95% confidence intervals (sample sizes shown in light gray). The mean of all departures was generated from one model with the three species, whereas the mean values for each of the three departure strategies were generated from a model with the three strategies and two species (mallard and gadwall). Broods that left the nest the same day as hatch and before the next dawn are shown in blue, whereas broods that left after the next dawn are shown in yellow, and broods that left the nest after two dawns are shown in red. This study took place at the Grizzly Island Wildlife Area (California, USA; 38.141°N, 121.970°W), within Suisun Marsh, 2015–2017

The mean length of time that ducklings remained at the nest after the first duckling hatched did not differ by species (*F*_2,49_ = 2.37, *p* = 0.10; Figure 2). Ducklings remained at the nest for 4.3–41.8 hr after the first duckling hatched before leaving the nest (*n* = 52 nests). After all the ducklings were determined to have hatched, mallard and gadwall ducklings remained at the nest for 7.2–38.7 hr (*n* = 26 nests). For nests that hatched ≥6 eggs (6–13 eggs; *n* = 23 nests), the mean ± *SD* of the hatch duration from the first duckling to emerge to the last duckling to emerge was 3.4 ± 2.3 hr.

**Figure 3:**
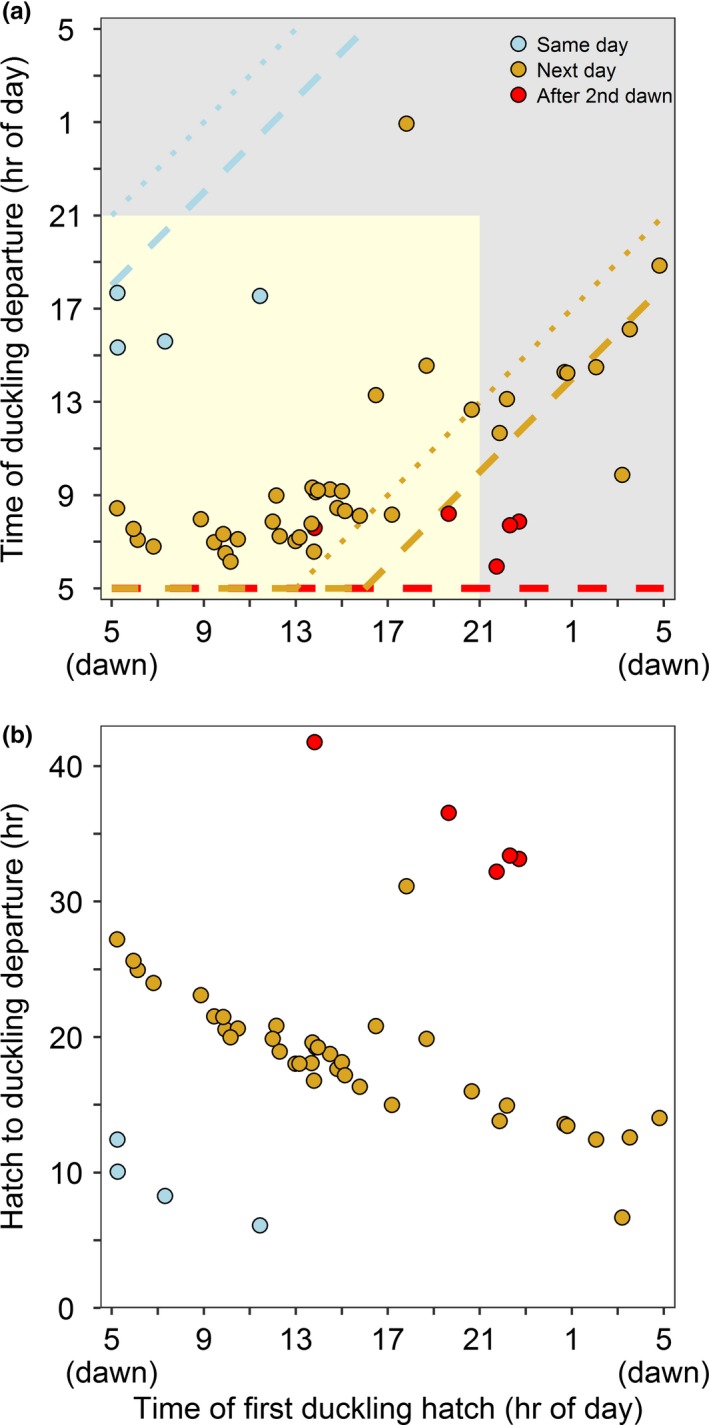
(a) The time of day of the first duckling to hatch (first duckling fully emerged from the egg) related to the time of day that mallard (*Anas platyrhynchos*) and gadwall (*Mareca strepera*) ducklings departed the nest (*n* = 47 nests). Daylight (day and twilight) is shaded in yellow, and night is shaded in gray. All times were standardized so that 05:00 hr local time is dawn in this figure, although statistics were conducted using time relative to nautical dawn. Broods that left the nest the same day as the start of hatch and before the next dawn are shown in blue, whereas broods that left the nest after the next dawn are shown in yellow, and broods that left the nest after two dawns are shown in red. The dashed lines, color coded to correspond with the three departure strategies, indicate the timing of a potential 13‐hr threshold for imprinting relative to the start of hatch, and the stippled lines indicate a 16‐hr threshold for imprinting (Hess, 1959). Birds departing above/to the left of the line departed after the imprinting period relative to the start of hatch and those departing below/to the right departed prior to the end of the critical period for imprinting. (b) The time of day of the first duckling hatch related to the duration of time spent at the nest, color coded the same as in panel (a). This study took place at the Grizzly Island Wildlife Area (California, USA; 38.141°N, 121.970°W), within Suisun Marsh, 2015–2017

**Figure 4:**
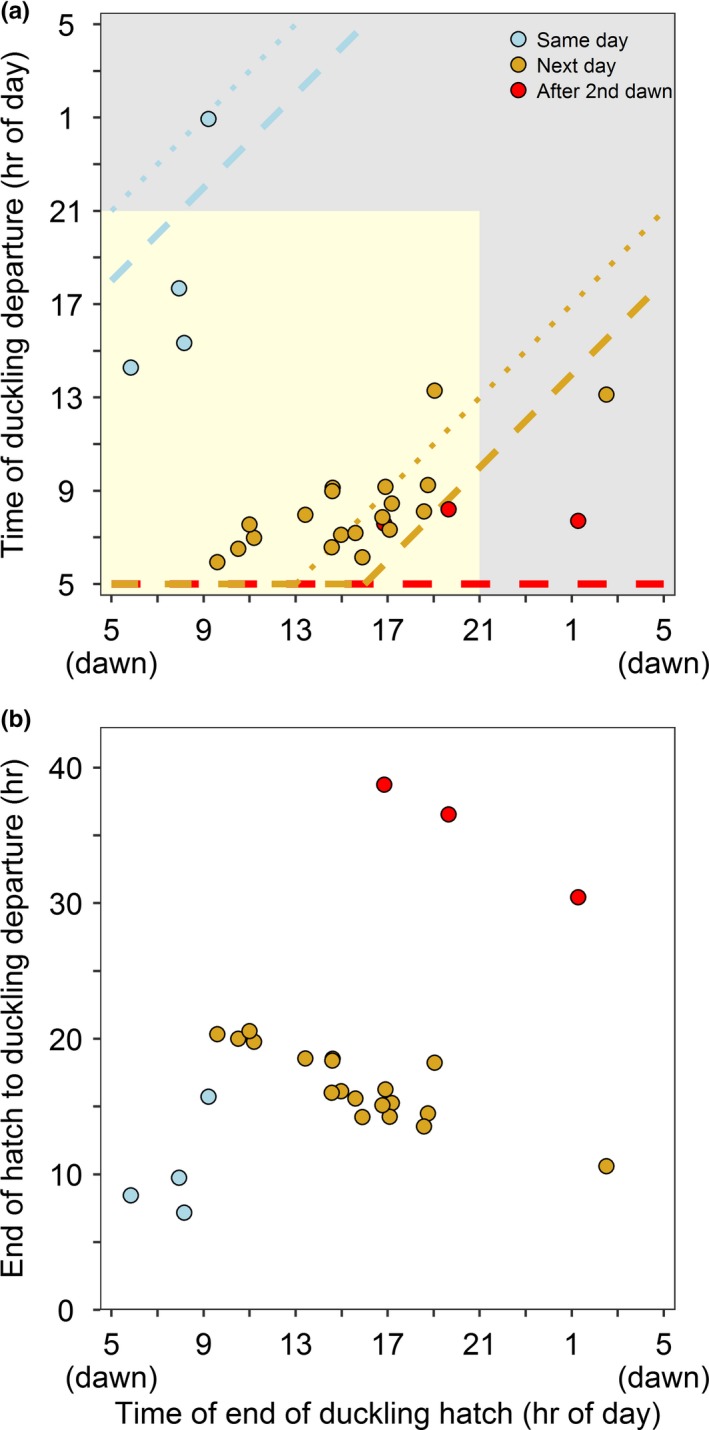
(a) The time of day of the end of hatch (last duckling fully out of the egg) related to the time of day that mallard (*Anas platyrhynchos*) and gadwall (*Mareca strepera*) ducklings departed the nest (*n* = 26 nests). Daylight (day and twilight) is shaded in yellow, and night is shaded in gray. All times were standardized so that 05:00 hr local time is dawn in this figure, although statistics were conducted using time relative to nautical dawn. Broods that left the nest the same day as the end of hatch and before the next dawn are shown in blue, whereas broods that left the nest after the next dawn are shown in yellow, and broods that left the nest after two dawns are shown in red. The dashed lines, color coded to correspond with the three departure strategies, indicate the timing of a potential 13‐hr threshold for imprinting relative to the end of hatch, and the stippled lines indicate a 16‐hr threshold for imprinting (Hess, 1959). Birds departing above/to the left of the line departed after the imprinting period relative to the start of hatch and those departing below/to the right departed prior to the end of the critical period for imprinting. (b) The time of day of the end of duckling hatch related to the duration of time spent at the nest, color coded the same as in panel (a). This study took place at the Grizzly Island Wildlife Area (California, USA; 38.141°N, 121.970°W), within Suisun Marsh, 2015–2017

For mallard and gadwall nests (*n* = 47), we identified three strategies of departure from the nest based on the timing of departure relative to the start of hatch (Figure 2). First, 9% of broods left the nest during daylight on the same day the first duckling was observed hatched (*n* = 4). These ducklings started to hatch between 0.3 and 6.4 hr after dawn and departed the nest 6.1–12.4 hr later. Second, 81% of broods left the nest after dawn the next day after the start of hatch (*n* = 38). For these ducklings, we observed a threshold where the timing of duckling departure changed markedly based on when the eggs began to hatch. For nests with ducklings that started to hatch earlier than ~16:00 hr standardized local time (11 hr after dawn in our study area) and departed the next day (*n* = 25), 100% of broods departed with the onset of daylight on the second day, a mean 2.8 hr after dawn (range 1.1–4.3 hr after dawn; Figure 3). For nests that started to hatch later in the day or at night, after ~16:00 hr local time, and departed the next day during daylight hours (*n* = 12), excluding the one brood that left the nest at night, ducklings departed a mean 8.4 hr after dawn. Third, 10% of broods departed the nest after the second dawn after the start of hatch (*n* = 5; Figure 3). All of these ducklings that waited until after the second dawn (32.2–41.8 hr after the start of hatch) departed the nest with the onset of daylight, within 0.9–3.2 hr of dawn. When we examined a subset of mallard and gadwall nests that had an estimate for the end of hatch (*n* = 26), 15% of ducklings left the nest the same day that the eggs finished hatching (10.3 hr after the end of hatch; range 7.2–15.7 hr), 73% left the nest after dawn the next day (16.6 hr after the end of hatch; range 10.6–20.6 hr), and 12% left the nest after the second dawn (35.2 hr after the end of hatch; range 30.4–38.7 hr) (Figure 4).

### 3.2 Egg and duckling predation

Predators killed ducklings at 15% of nests after hatch (*n* = 8 of 55 nests) and depredated eggs at 10% of nests during the ~2‐day period prior to hatch (*n* = 6 of 60 nests); the risk of depredation during these ~2‐day periods was not statistically different (*χ* ^2^ = 0.21, *df* = 1, *p* = 0.65). It should be noted, however, that the time windows for this comparison are not completely comparable because most ducklings did not remain at the nest for 2 full days after hatch and therefore the after‐hatch time frame was smaller than the before‐hatch time frame that was compared. Additionally, all predation bouts on hatched nests occurred within the first day after hatch. Furthermore, there was no way to determine exactly when predation occurred relative to the timing of hatch for nests that were fully depredated as eggs and therefore did not hatch.

For nests that were depredated in the final 2 days of incubation before hatch (*n* = 6), only one nest contained eggs that survived to hatch, and this nest was visited again by a striped skunk (*Mephitis mephitis*) after hatch, which caused the hen to immediately leave the nest with her ducklings. All of the eggs in the other five nests were depredated by striped skunk (*n* = 2 nests), raccoon (*Procyon lotor*; *n = *2 nests), and common raven (*Corvus corax*; *n* = 1 nest).

Predation bouts on ducklings occurred between 2.7 and 21.5 hr after the start of hatch (11.6 ± 6.2 hr). A coyote (*Canis latrans*) and an avian predator that visited one nest sequentially were responsible for killing 11 of 12 ducklings, and the hen also was killed (off camera) at that nest. Another nest had 8 of 8 ducklings depredated by a striped skunk. At the other six nests with dead ducklings, one nest had 2 of 6 ducklings killed by a gopher snake (*Pituophis catenifer*) and then two more killed by an avian predator; three nests had 2 of 6 ducklings, 2 of 8 ducklings, and 2 of 10 ducklings killed by gopher snakes; one nest had 2 of 12 ducklings killed by an unknown predator; and one nest had 2 of 11 ducklings crushed and killed, but not eaten, by Tule elk (*Cervus canadensis nannodes*). Additionally, potential predators were observed on camera at an additional 7% of hatched nests (*n* = 4 nests) but did not kill any ducklings at those nests. Multiple predator species were observed at the same nest for 25% (*n* = 3) of nests that were visited by a predator at least once (*n* = 12). The same predator species (or the same individual predator) was observed repeatedly over multiple bouts at two nests, and one nest had two snakes observed at the same time.

Of the 12 nests where a potential predator appeared on camera, only once did the hen and ducklings immediately flee the nest (from a striped skunk). Instead, the ducklings remained at the nest for 1.5–18.6 hr after the last time a predator was seen on camera at the nest (9 of 12 nests). At the other two nests, either the hen or all the ducklings were killed, and therefore, there was no time of departure available to be calculated. The range of times that hens remained at the nest after a predator encounter was comparable, irrespective of whether the predator killed any ducklings. When the potential predator did not kill any ducklings (*n* = 4), ducklings left the nest 0–18.6 hr after the potential predator was last at the nest; at one nest, the hen and hatched ducklings fled immediately from a striped skunk, and at the other three nests, gopher snakes did not consume any ducklings. When some ducklings, but not the entire brood, were killed by a predator (*n* = 6), the remaining ducklings departed the nest 1.5–16.9 hr after the predator left the nest, suggesting that the presence of a predator killing ducklings was not enough impetus to cause the hen to leave the nest immediately with her ducklings as a direct result of the predator.

## 4 DISCUSSION

Minimizing time at a nest after hatch may decrease the risk of predation on ducklings at the nest, although female ducks may weigh this risk against the likelihood of encountering predators while they are moving to wetlands (Chouinard & Arnold, 2007; Mauser et al., 1994b) and the time constraints on ducklings to be developmentally ready to leave the nest (Fabricius, 1964). For example, if a brood is fully hatched during the night or early in the day, then a hen may elect to leave the nest quickly once there is daylight to shorten the window for an encounter with a diurnal predator (e.g., snakes, ravens) while the brood is still in the nest. However, leaving the nest quickly may result in ducklings departing the nest prior to the critical period of imprinting and before their motor coordination is fully matured (Fabricius, 1964). If a brood is not fully hatched until late afternoon or early evening, then a hen may choose to remain sheltered at the nest overnight to avoid encountering nocturnal predators in the dark while en route to water, and leave the nest the next day when temperatures are warmer and her ducklings are more developmentally ready to depart the nest.

The main mammalian predators of duck eggs and ducklings in our study area (striped skunk, raccoon, and coyote) are most active at night, whereas gopher snakes and avian predators are most active and visit duck nests during daylight (Croston et al., 2018). In the present study, all but one brood departed the nest during daylight hours, which is consistent with avoiding temporal overlap with mostly nocturnal mammalian predators while en route to wetlands. Furthermore, the majority of hens departed the nest with their broods just after dawn (2–3 hr after dawn) to begin their journey to brood water. Transiting to wetlands early during the day, in addition to minimizing the risk of contact with nocturnal predators, also may minimize the risk of predation by gopher snakes, which tend to forage diurnally and visit bird nests later in the morning and throughout the afternoon after temperatures warm (Croston et al., 2018; Degregorio et al., 2014; Lockyer, Coates, Casazza, Espinosa, & Delehanty, 2013).

The proportion of nests with depredated ducklings was substantial, with 15% of hatched nests experiencing loss of at least one duckling prior to departure from the nest. Although sound and movement associated with the presence of ducklings at the nest did not appear to increase the likelihood of predation when compared with eggs prior to hatch (10% of nests), low power due to our sample size may have made it difficult to detect a difference between these groups. Furthermore, all predation on ducklings occurred within the first 24 hr after hatch and the time window that we examined for predation on eggs may have been larger because we selected a time window of ~2 days but were unable to determine exactly when the predation occurred relative to hatch (as 5 of 6 depredated nests were fully consumed by predators and did not end up hatching). Although daily nest survival generally increases with nest age (Garrettson & Rohwer, 2001; Klett & Johnson, 1982), it is possible that increased noise and vocalizations from the embryo in the day or so prior to hatch (Afton & Paulus, 1992) may have allowed these nests to be more detectable by predators. Additionally, the type of predator observed depredating eggs versus ducklings appeared to differ. Gopher snakes depredated ducklings at four nests but did not depredate any eggs, supporting previous observations where gopher snakes attempted to but were unsuccessful at consuming mallard and gadwall eggs (Croston et al., 2018). Additionally, striped skunks and raccoons depredated eggs at five nests in the 2 days prior to hatch. Striped skunks killed ducklings at two nests although raccoons were not observed depredating ducklings. Avian predators were observed depredating both eggs (*n* = 1 nest) and ducklings (*n* = 2 nests). Surprisingly, 100% of hens at nests where predators depredated ducklings but did not kill the entire brood (*n* = 7 depredation bouts at six nests) did not leave the nest with their ducklings until a mean 7.0 hr (range 1.5–16.9 hr) after the departure of the predator, despite 3 of these depredation bouts occurring during daylight between 12:00 and 14:00 hr. Most predation bouts in which some but not all ducklings were killed involved gopher snakes. Incubating hens return to their nests quickly after being flushed by gopher snakes (mean 15 min), but wait longer to return when flushed by striped skunk or raccoon (Croston et al., 2018). This suggests that hens do not view gopher snakes as a direct threat to themselves and may also explain the hens' choices to remain at the nest with their remaining ducklings until the ideal time for departure, despite the fact that some of their ducklings were depredated.

Although mallard and gadwall are nidifugous, ducklings cannot leave the nest immediately and development constrains the minimum time needed at the nest between the start of hatch and departure from the nest. First, the brood needs to hatch, which we observed to take 3 (±2) hours for a subset of nests that hatched ≥6 eggs, and another study observed hatch to take 3–8 hr for the whole brood (Bjärvall, 1967). For each duckling, the drying of feathers and physical maturation of motor coordination to be able to leave the nest can happen in parallel with imprinting upon the hen, and the clock for these developmental trajectories starts for each individual duckling when they hatch. Thus, part of a brood may be ready to depart the nest before the rest. A hen may wait for all of her ducklings to make it through the critical period of imprinting between 13 and 16 hr after hatch (Bjärvall, 1967; Fabricius, 1964; Hess, 1959), or she may leave the nest after some of her ducklings have imprinted (Boyd & Fabricius, 1965) and as soon as they are physically able to leave the nest, assuming that social facilitation will promote the brood to stick together as a group and follow her (Fabricius, 1964; Hess, 1959).

Duckling departure from nests occurred primarily during the early morning, as demonstrated in other studies on the same species (Bjärvall, 1968; Girard, 1941; Krapu et al., 2006). The narrow window of peak departure times from the nest for these ducklings (2–3 hr after dawn) suggests that this is the preferred timing for nest exodus, although there may be specific events that influence this timing. We observed that hatch during daylight hours (prior to ~16:00 hr local time) appeared to be the time window that minimized the period ducklings were at the nest and consequently the length of time that ducklings would be vulnerable to nest predators, while still allowing ducklings to become physically ready to leave the nest and undergo the critical period of visual and auditory imprinting prior to departure the next morning just after dawn. Hens with nests that started and finished hatch later in the day (after ~16:00 hr) were likely constrained by the developmental needs of their ducklings and tended to remain at the nest overnight and leave the next morning either at the preferred time or later in the day.

We observed three unique departure strategies for mallard and gadwall hens. Mallard and gadwall hens that elected to leave the nest with their ducklings in the afternoon on the same day as the start of hatch (9%) may have elected to leave as soon as the ducklings were dry and physically able to, regardless of whether they had yet fully imprinted (Bjärvall, 1967; Fabricius, 1964; Hess, 1959), as long as this occurred during daylight. The most common departure strategy (81% of broods) was for ducklings to leave the nest after dawn on the day after hatch began, and all but one of these broods departed the nest during daylight hours. Of these broods, 95% left the nest >13 hr after hatch began, which would have allowed enough time at the nest for at least the first hatched duckling in the brood to fully imprint on the hen. However, within this strategy, we observed that 100% of nests starting to hatch prior to ~16:00 hr local time departed within the first 4.3 hr after dawn the next day. In contrast, 75% of nests that started to hatch later in the day or at night (after 16:00 hr) left the nest more than 8 hr after dawn the next day. The third departure strategy was comprised of broods that started to hatch after ~16:00 hr on day one and departed the nest within the first 3.2 hr after the second dawn (10%). These ducklings would have had ample time to both physically mature and imprint on the hen (Fabricius, 1964), yet they remained on the nest longer. The narrow window of departure times even for these nests supports that this early‐morning timing for nest departure is the preferred time window for nest exodus because these hens were not limited by impending night and presumably could leave the nest at any time.

As duckling broods begin to hatch, multiple developmental changes happen simultaneously, making it a challenge to ascertain if one aspect is more or less important in determining the timing of duckling departure from the nest. The extended brooding period for some nests may have resulted from developmental differences among duckling broods or other factors not accounted for in this study. It may have taken longer for some broods to hatch than other broods that began hatch earlier, and therefore, they needed more total time for the ducklings to dry and be physically able to leave the nest. Additionally, these hens may have chosen to wait longer at the nest after hatch than other broods that began hatch earlier, to allow more of their ducklings to make it through the critical period of imprinting prior to departure from the nest. Some eggs may fail to hatch, causing the hen to wait at the nest for a longer period before leaving the nest. We visually inspected our data for potential relationships between departure strategy and aspects of clutch size, including the number of eggs that hatched or failed to hatch, and we did not detect any trends. Additionally, the length of time that we observed to hatch the entire brood should not have delayed departure from the nest for these broods that began hatch later in the day, based on our observations of hatch duration. What we did observe, however, were multiple nests with broods that were fully hatched by early afternoon (~13:00 hr) and remained at the nest until the next morning. If predation risk to the ducklings at the nest was the most important factor in the hen's decision regarding when to leave the nest (i.e., more important than predation risk to the ducklings off the nest), we would have expected more broods to have left the nest on the same day that hatch ended instead of waiting at the nest for an additional night prior to leaving the nest. With daylight lasting until at least 19:50 hr local time, there should have been ample time for ducklings that hatched prior to 13:00 hr to dry and be physically able to leave the nest with the hen prior to darkness, although this decision would have come at the expense of imprinting on the hen.

As hens lead their ducklings from the nest toward water, their ultimate success may be determined by a combination of nest departure time, proximity and ease of travel to good aquatic habitat, and whether the ducklings have fully imprinted on the hen. Whether hens are successfully able to lead their ducklings to nearby water could be affected by the timing of nest departure and the local habitats that are available in reasonable proximity to a given nest, which we did not examine in the present study. Previous studies have observed a range of distances that ducklings had to travel initially from their nests to water. Mauser et al. (1994b) observed ducklings traveling up to 200 m in northern California, whereas Amundson and Arnold (2010) observed ducklings traveling up to 2000 m in North Dakota, Cowardin et al. (1985) found ducklings traveling up to 3,780 m in North Dakota, and Chouinard and Arnold (2007) observed ducklings traveling up to 4,560 m in the San Joaquin Valley of California. Ducklings in the present study that left the nest the same day that hatch began were more limited in the distance they could relocate during daylight hours; thus, the availability of suitable wetland habitats may have been limited for that day. For ducklings that leave in the early‐morning hours, regardless of when they hatched, there is more daylight during which to transit; therefore, more suitable wetland habitat is likely to be accessible before nightfall. There is some evidence from the behavior of GPS‐marked hens that they may predetermine where they will take their brood, and thus, may be able to assess the distance of their journey to brood habitat (USGS unpublished data).

Our study indicates a preferred time for broods to depart from nests (2–3 hr after dawn) and suggests that there may be an ideal time window for nests to hatch (daylight prior to 16:00 hr) that minimizes time at the nest and consequently minimizes predation risk. The ideal time window for hatch allows enough time for all ducklings in the brood to hatch and dry, allows ducklings to become physically ready to leave the nest and imprint on the hen prior to departure from the nest, and allows ducklings to depart the nest at the preferred early‐morning time. The timing of departure from the nest, imprinting on the hen, predation risk at the nest, and predation risk while traversing from nest sites to brood water are all likely important and interacting components that influence duckling survival.

## CONFLICT OF INTEREST

None declared.

## ETHICS STATEMENT

Research was conducted with the approval of the U.S. Geological Survey Western Ecological Research Center's Animal Care and Use Committee.

## AUTHOR CONTRIBUTIONS

J.T.A., M.L.C., and C.F. contributed substantial resources and initiated the project. S.H.P., J.T.A., M.P.H., C.A.H., R.C., and M.L.C. conceived the idea and designed the study. S.H.P., J.T.A., and R.C. developed and designed the methods. S.H.P., J.T.A., M.L.C., M.P.H., and C.A.H. collected the data. S.H.P., M.P.H., and R.C. curated the data; S.H.P. statistically analyzed the data. S.H.P. wrote the paper, and all authors edited the paper.

## Data Availability

The data in this article are in ScienceBase: https://doi.org/10.5066/P93ZFTZI.
